# A New Mucilage

**Published:** 1877-12

**Authors:** 


A New Mucilage.—The *Journal de Pharmacie* states that if, to a solution of gum-arabic, measuring 8⅓ fluid ounces, a solution of 30 grains of sulphate of aluminum, dissolved in two-thirds of an ounce of water, be added, a very strong mucilage is formed, capable of fastening wood together, or of mending porcelain or glass.

